# Real-World Efficacy and Toxicity of Ipilimumab and Nivolumab as First-Line Treatment of Metastatic Renal Cell Carcinoma (mRCC) in a Subpopulation of Elderly and Poor Performance Status Patients

**DOI:** 10.3390/cancers17030522

**Published:** 2025-02-04

**Authors:** Noa Shani Shrem, Ana-Alicia Beltran-Bless, Sunita Ghosh, Camilla Tajzler, Lori A. Wood, Christian Kollmannsberger, Naveen S. Basappa, Jeffrey Graham, Nazanin Fallah-Rad, Daniel Y.C. Heng, Denis Soulières, Aly-Khan A. Lalani, Rodney H. Breau, Antonio Finelli, Simon Tanguay, Bimal Bhindi, Georg Bjarnason, Frederic Pouliot, Christina Canil

**Affiliations:** 1 The Legacy Heritage Oncology Center & Dr. Larry Norton Institute, Soroka University Medical Center, Beer Sheva 84101, Israel; 2 Division of Medical Oncology, University of Ottawa, Ottawa, ON K1H 8M5, Canada; 3 Faculty of Medicine and Dentistry, University of Alberta, Edmonton, AB T6G 2R3, Canada; 4 Centre of Innovative Medicine, Research Institute—McGill University Health Centre, Montreal, QC H3H 2R9, Canada; 5 Capital Health Queen Elizabeth II Hospital, Halifax, NS B3H 2Y9, Canada; 6 BC Cancer, Vancouver, BC V3V 1Z2, Canada; 7 Cross Cancer Institute, University of Alberta, Edmonton, AB T6G 2R3, Canada; 8 CancerCare Manitoba, Winnipeg, MB R3E 0V9, Canada; 9 University Health Network, Toronto, ON M5G 2C4, Canada; 10 Alberta Health Services, Calgary, AB T5J E34, Canada; 11 Centre Hospitalier de l’Université de Montréal, Montreal, QC H2X 0A9, Canada; 12 Juravinski Cancer Centre, McMaster University, Hamilton, ON L8V 5C2, Canada; 13 Division of Urology, Department of Surgery, University of Ottawa, Ottawa, ON K1H 8M5, Canada; 14 McGill University Health Centre, Montreal, QC H4A 3J1, Canada; 15 Sunnybrook Health Sciences Centre, Toronto, ON M4N 3M5, Canada; 16 Centre Hospitalier Universitaire de Québec, Quebec, QC G1R 2J6, Canada

**Keywords:** metastatic renal cell carcinoma, ipilimumab and nivolumab, elderly, poor performance status, low KPS, efficacy, toxicity, real-world evidence

## Abstract

Background: Ipilimumab and nivolumab (ipi/nivo) improved overall survival (OS) compared to sunitinib in the pivotal Checkmate 214 trial of metastatic renal cell carcinoma (mRCC) with International Metastatic RCC Database Consortium (IMDC) intermediate/poor risk disease. We evaluated the efficacy and toxicity of ipi/nivo in older and frailer populations in a real-world mRCC cohort. Methods: Analysis was conducted on a real-world cohort with mRCC (N = 551) treated with first-line ipi/nivo from the Canadian Kidney Cancer information system (CKCis) database from January 2014 to December 2021. A comparison was made between outcomes and toxicity in patients 1. <70 versus (vs.) ≥70 yo, 2. <75 vs. ≥75 yo, and 3. KPS ≥70 vs. <70 yo. OS, progression-free survival (PFS), and time to treatment failure (TTF) were calculated by Kaplan–Meier analysis. Log-rank tests were used for comparison between groups. Results: Ipi/nivo treatment had no impact on survival outcomes or toxicity for patients >70 yo and >75 yo when controlled for IMDC. However, when comparing patients with KPS > 70 vs. KPS < 70, patients with a poor performance status had decreased median OS at 54.5 m vs. 10.8 m (*p*-value < 0.0001) and PFS at 11.6 vs. 3.1 m (*p*-value < 0.0001). Conclusions: The use of ipi/nivo in mRCC demonstrated similar survival outcomes and toxicity in an older patient population. In patients with a poor performance status, it was associated with inferior OS and PFS. We believe that ipi/nivo is a reasonable treatment option for these patient populations, particularly in older patients.

## Simple Summary

The results of a large, randomized control trial demonstrated improved survival and quality of life with ipilimumab and nivolumab immunotherapy compared to oral targeted therapy with sunitinib. Elderly patients and those with poor performance status are often excluded from clinical trials despite comprising a significant proportion of patients seen in the clinical setting. As a result, there is limited information to guide treatment for these patient groups. Our study analyzed real-world patient data of 551 patients with metastatic renal cell cancer from the Canadian Kidney Cancer information system (CKCis) database to assess the clinical outcomes of ipilimumab and nivolumab for older and less fit patients compared to younger and more fit cohorts. We found no difference in outcomes for elderly compared to younger patients. However, patients with poor performance status had poorer overall survival and shorter response to treatment compared to more fit patients.

## 1. Introduction

The results of the phase III Checkmate 214 trial prompted the combination of dual immunotherapy agents ipilimumab and nivolumab to become a new standard of care for the first-line treatment of metastatic clear-cell renal cell carcinoma patients with intermediate and poor risk disease based on the International Metastatic RCC Data Consortium (IMDC) risk groups [1,2,3]. The combination of ipilimumab and nivolumab was shown to have improved overall survival (OS) with higher response rates than the prior standard of care sunitinib, a vascular endothelial growth factor tyrosine kinase inhibitor, but it was also associated with a significant risk of immune-related toxicities.

Most individuals who participate in these clinical trials are often not representative of the general population. These study participants must satisfy stringent eligibility criteria, including good physical fitness and excellent end organ function with minimal comorbidities.

Elderly patients and those with poor performance status are generally excluded from clinical trials despite comprising a growing proportion of patients seen in clinical settings [4,5]. Most patients diagnosed with renal cell carcinoma are aged over 65, with one-quarter being over 75 years of age. However, only approximately one-third of patients included in clinical trials of metastatic renal cell carcinoma are over 65 years of age [6,7]. In the Checkmate 214 study, a subgroup analysis in patients greater than 65 years old favored doublet immunotherapy over sunitinib; however, the confidence intervals were wide as the patient numbers were small, particularly in the greater than 75 years old subgroup [1]. In addition, many clinical trials of systemic therapy prohibit patients with poor performance status such as Karnofsky Performance Status (KPS) < 70 from enrolling in studies [8]. Elderly patients and those with poor performance status are a heterogeneous group with a higher burden of health conditions and medication use, as well as differences in pharmacokinetics and pharmacodynamics that could alter drug profiles, which makes the extrapolation of clinical trial results challenging [9]. There is a common belief among physicians that systemic treatment is likely not as well tolerated in elderly patients or those with poor performance status due to medical comorbidities and frailty. The International Society of Geriatric Oncology has stated that age should not be a barrier to effective treatment [7].

Due to this lack of representation in clinical trials, limited data are available regarding the efficacy and toxicity of ipilimumab and nivolumab in elderly patients and those with poor performance status [10,11,12,13]. Real-world evidence, that is, data collected in the every-day practice setting, can generate outcomes to compliment the knowledge gained from traditional phase III clinical trials to better guide the treatment of these underrepresented populations [14].

The aim of this study was to analyze real-world patient data from the Canadian Kidney Cancer information system (CKCis) database and determine the efficacy, measured as overall survival (OS), progression-free survival (PFS), time to treatment failure (TTF), overall response rate (ORR), and toxicity of ipilimumab plus nivolumab as a first-line treatment in RCC in the subpopulation of the elderly (age of ≥70 years, age of ≥75 years) and poor performance status (KPS < 70) patients in comparison to a younger, relatively well-performing patient population.

## 2. Materials and Methods

### 2.1. Study Design

The Canadian Kidney Cancer information system (CKCis) is a web-based national database that contains pertinent retrospective as well as prospective de-identified patient data collected from consented patients who have been diagnosed and treated for renal cell carcinoma since 2011. It includes fifteen Canadian centers across six provinces [15]. This study included a real-world cohort of (N = 551) patients from the CKCis database with metastatic renal cell carcinoma that were treated with first-line ipilimumab and nivolumab between January 2014 and December 2021. This database included 78.4% clear-cell and 17.8% non-clear-cell renal cell carcinoma patients. Data were restricted to patients who started systemic treatment on or before 2021 to allow for 2 years of follow-up (31 December 2023). Patients were excluded if they received prior treatment with immunotherapy.

Patients were stratified according to age (equal to and above 70 years of age, less than 70 years of age, equal to and above 75 years of age, less than 75 years of age) and performance status (KPS less than 70 and KPS equal to and above 70). A comparison was made between patients aged <70 years old vs. ≥70 years old, between patients age <75 years old vs. ≥75 years old, and KPS ≥70 vs. <70.

Age was treated as a dichotomous variable and divided into two cutoffs (>0 vs. >75 years of age) for ease of interpretation and extrapolation in the clinical setting. These two cutoffs were decided based on geriatric oncology experts and international organizations recommendations [16,17,18].

Baseline demographic, clinical, and laboratory characteristics were extracted, including sex, prior nephrectomy or metastasectomy, metastatic sites, and IMDC risk group at baseline. The number of ipilimumab and nivolumab cycles received, the reason for discontinuing treatment, and subsequent lines of treatment were collected. Data on toxicity, grade, and type according to the Common Terminology Criteria for Adverse Events (CTCAE) 4.03 were collected as well.

### 2.2. Outcomes of Interest and Statistical Analysis

Mean and standard deviation (SD) were reported for continuous variables, and frequency and proportions were reported for categorical variables. Chi-square tests were used to compare two categorical variables. An independent *t*-test of proportions was used to compare individual proportions. Toxicity was graded as per CTCAE v4.03 or any toxicity that resulted in a dose/schedule change. Overall survival was calculated from the time to start of systemic treatment to the date of death from any cause, subjects alive at the last date of follow-up were censored. Progression-free survival (PFS) was calculated from the start of systemic treatment to disease progression or death of any cause whichever occurred first. Subjects who did not have a date of disease progression and were alive at the last date of follow-up were censored from the analysis. Time to treatment failure (TTF) was calculated from the start of systemic treatment to date of discontinuation of treatment or death due to any cause. Time to survival was calculated for OS, PFS, and TTF and Kaplan–Meier estimates, and the 95% confidence intervals were reported and compared using log-rank statistics. Overall response rate (ORR) was defined as the best measured response at the local site and categorized as complete response (CR), partial response (PR), stable disease (SD), or progressive disease (PD). ORR was defined as complete response or partial response, and ORR was compared between the groups using Chi-square tests. Disease control rate was defined as a composite of ORR and stable disease. Cox’s regression analysis was conducted for OS, PFS, and TTF, adjusting for IMDC risk and hazard ratio (HR), and the corresponding 95% confidence intervals were reported. Survival data for cause-specific survival for patients who died of renal cell cancer were also computed and compared as available. A *p*-value of <0.05 was considered statistically significant. SAS version 9.4 (SAS Institute Inc., Cary, NC, USA) was used for all the statistical analysis.

## 3. Results

### 3.1. Overall Cohort

There were 551 patients included in the total cohort. The median age was 63.4 years (range 26.6–91.0). In this cohort, the majority of patients were male at 73.9% compared to female at 26.1%. Median follow-up was 32.1 months (range 0.53–117.16 months). There were 265 events where events are defined as patients who died, with 286 patients still alive on the last date of follow-up.

### 3.2. Comparing Age <70 with Age ≥70 Years Old

#### 3.2.1. Patient Characteristics

In total, 415 patients (75%) were included in the age <70 group and 136 (25%) were included in the age ≥70 group, with median ages of 60.6 years and 74.4 years, respectively. Sex distribution was similar in the two groups. Fewer older patients completed four cycles of ipilimumab and nivolumab (60/136, 44.1%) when compared to younger patients (250/415, 60.2%). Patients were more likely to have undergone surgical resection (nephrectomy and metastasectomy) if they were younger (Table 1).

#### 3.2.2. Outcomes

**Figure 1:**
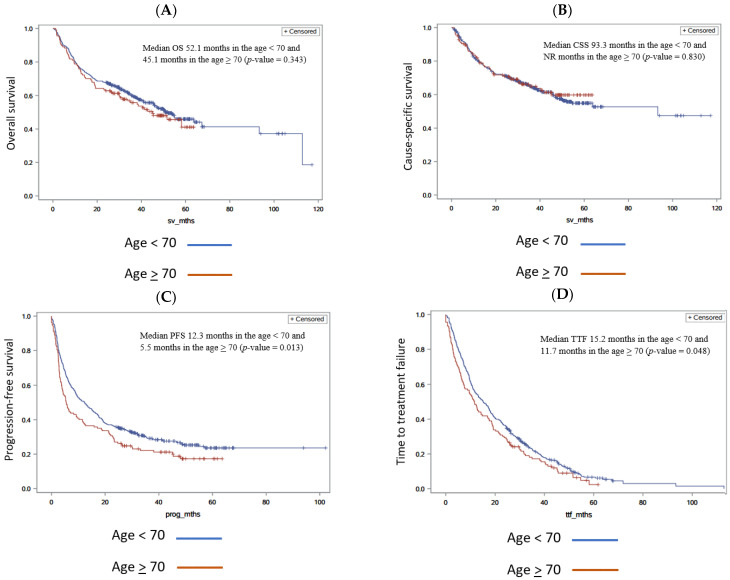
Comparisons of outcomes for age <70 and age >70. Kaplan–Meier curves for age <70 and age >70. (**A**) Overall survival comparing age <70 and age >70, (**B**) cause-specific survival comparing age <70 and age >70, (**C**) progression-free survival comparing age <70 and age >70, and (**D**) time to treatment failure comparing age <70 and age >70. NR = not reached.

The median OS was 52.1 months in the age <70 group and 45.1 months in the age ≥70 group (*p*-value 0.343 in univariate analysis, 0.352 multivariate) (Figure 1A). Median cause-specific survival (CSS) due to renal cell carcinoma was 93.3 months in the age <70 group and not reached in the age ≥70 group (*p*-value 0.830 in univariate analysis, 0.897 in multivariate analysis) (Figure 1B). The median PFS was 12.3 months in the age <70 group and 5.5 months in the age ≥70 group (*p*-value 0.013 in univariate analysis, 0.019 in multivariate analysis) (Figure 1C). The median TTF was 15.2 months vs. 11.7 in the age <70 group and the age ≥70 group, respectively, (*p*-value 0.048 in the univariate and 0.077 in the multivariate analysis) (Figure 1D). Response rates were similar in both groups, with a disease control rate of 62.2% (229/368) in the age <70 group compared to 60.7% (68/112) in the age ≥70 group (Table 1).

#### 3.2.3. Toxicity

The toxicity rates were similar in the two populations (39.3% in the age <70 and 39.7% in the age ≥70 groups) (*p*-value 0.0929). The discontinuation rates due to toxicity were 36.1% (97/269) in the age <70 group and 52.6% (51/97) in the age ≥70 group. The most common toxicity in both groups was colitis. There was one death due to myocarditis in the age ≥70 group.

#### 3.2.4. Second-Line Treatment

Fewer older patients received second-line treatment (51/136, 37.5%) compared to younger patients (201/415, 48.4%) (*p*-value 0.026). The most common second-line treatment was sunitinib in both groups.

### 3.3. Comparing Age <75 with Age ≥75 Years Old

#### 3.3.1. Patient Characteristics

In total, 494 patients (90%) were included in the age <75 group and 57 (10%) in the age ≥75 group. The patients’ median age was 61.9 years in the age <75 group and 77.7 years in the ≥75 group. Sex distribution was comparable in the two groups (Table 2). More patients with age >75 had IMDC poor risk disease (18/48, 37.5% compared to 132/406, 32.5%). Also, the younger cohort was more likely to undergo nephrectomy (67.8% vs. 50.9%, respectively). Fewer older patients (≥75 years old) completed four cycles of ipilimumab and nivolumab (26/57, 45.6%) when compared to younger patients (284/494, 57.5%) (Table 2).

#### 3.3.2. Outcomes

**Figure 2:**
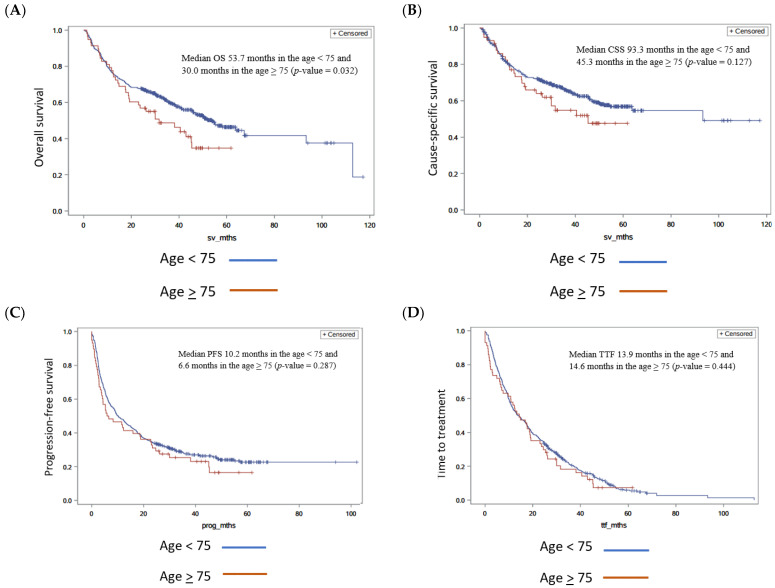
Comparisons of outcomes for age <75 and age >75. Kaplan–Meier curves for age <75 and age ≥75. (**A**) Overall survival comparing age <75 and age ≥75, (**B**) cause-specific survival comparing age <75 and age ≥75, (**C**) progression-free survival comparing age <75 and age ≥75, and (**D**) time to treatment failure comparing age <75 and age ≥75.

The median OS was 53.7 months in the age <75 group and 30.0 months in the age ≥75 group (*p*-value 0.032 in univariate and 0.157 in multivariate analysis) (Figure 2A). Median cause-specific survival was 93.3 months in the age <75 group and 45.3 months in the age ≥75 group (*p*-value 0.127 in univariate and 0.320 in multivariate analysis) (Figure 2B). The median PFS was 10.2 months in the age <75 group and 6.6 months in the age ≥75 group (*p*-value 0.287 in univariate and 0.386 in multivariate analysis) (Figure 2C). The median TTF was 13.9 months vs. 14.6 in the age <75 group and age ≥75 group, respectively, (*p*-values 0.444 and 0.469 in multivariate analysis) (Figure 2D). Response rates were similar in both groups, with disease control at 61.3% in the age <75 group (264/431) and 67.3% (33/50) in the age ≥75 group (Table 2).

#### 3.3.3. Toxicity

The toxicity rates were similar in patients aged <75 (195/494, 39.5%) and aged ≥75 (22/57, 38.6%) (*p*-value 0.90). The discontinuation rates due to toxicity were 38.9% (128/329) in the age <75 group and 54.1% (20/37) in the age ≥75 group. The most common toxicity in both groups was colitis. One patient in the age ≥75 group died of myocarditis.

#### 3.3.4. Second-Line Treatment

Second-line treatment was received in 47.4% of patients aged <75 (234/494) and 31.6% of patients aged >75 (18/57) (*p*-value 0.024). Again, sunitinib was the most common second-line therapy used in both groups.

### 3.4. Comparing KPS ≥70 with KPS <70

#### 3.4.1. Patient Characteristics

In total, 448 patients (88%) were included in the KPS ≥ 70 group and 63 (12%) in the KPS < 70 group. The patients’ median age was 63.4 years in the KPS ≥ 70 group and 64.7 years in the KPS < 70 group. Sex distribution was different in the two groups, with a higher percentage of males in the KPS > 70 group. There were more patients with KPS < 70 that had IMDC poor risk disease (42/52, 80.8%) compared to the KPS ≥ 70 group (107/395, 27.1%) (*p*-value < 0.0001), and fewer patients in the KPS < 70 group had nephrectomy. A total of 59% of patients in the KPS ≥ 70 group (266/448) completed four cycles of nivolumab versus 36.5% of patients with KPS ≤ 70 (23/63) (Table 3).

#### 3.4.2. Outcomes

**Figure 3:**
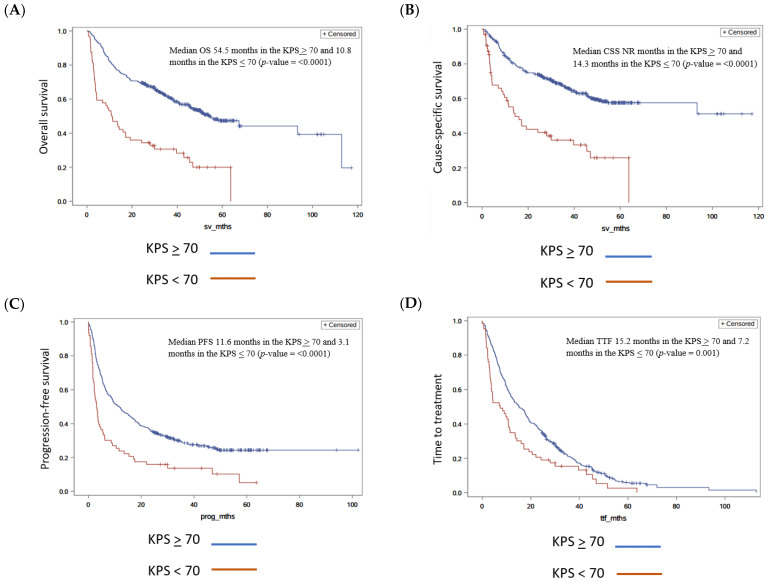
Comparisons of outcomes for KPS > 70 and KPS < 70. Kaplan–Meier curves for KPS < 70 and KPS > 70. (**A**) Overall survival comparing KPS < 70 and KPS > 70, (**B**) cause-specific survival comparing KPS < 70 and KPS > 70, (**C**) progression-free survival comparing KPS < 70 and KPS > 70, and (**D**) time to treatment failure comparing KPS < 70 and KPS > 70. NR = not reached.

The median OS was 54.5 months in the KPS ≥ 70 group and 10.8 months in the KPS < 70 group (*p*-value < 0.0001 in univariate analysis and 0.0009 in multivariate analysis) (Figure 3A). The median CSS due to metastatic renal cell carcinoma was not reached in the KPS ≥ 70 group and 14.3 months in the KPS < 70 group (*p*-value < 0.0001 in univariate and 0.009 in multivariate analysis) (Figure 3B).

The median PFS was 11.6 months in the KPS ≥ 70 group and 3.1 months in the KPS < 70 group (*p*-value < 0.0001 and 0.0007 in multivariate analysis) (Figure 3C). The median TTF was 15.2 months in the KPS ≥ 70 group and 7.2 months in the KPS < 70 group (*p*-value 0.001 in univariate analysis and 0.123 in multivariate) (Figure 3D). The response rates were similar in both groups, with disease control at 62.4% (246/394) in the KPS ≥ 70 group and 54.9% (28/51) in the KPS < 70 group (Table 3).

#### 3.4.3. Toxicity

The toxicity rates were higher in the KPS ≥ 70 group (184/448, 41.1%) than in the KPS < 70 group (16/63, 25.4%) (*p*-value 0.017). In the KPS ≥ 70 group, treatment discontinuation due to toxicity was 41.5% (122/294) and 40.4% (19/47) in the KPS < 70 group. One patient with KPS > 70 died of myocarditis.

#### 3.4.4. Second-Line Treatment

The rates of second-line treatment were different between the two groups, with 48.6% of patients with KPS ≥ 70 (218/448) and 28.6% of patients with KPS < 70 (18/63) (*p*-value 0.003). Sunitinib was the most common second-line treatment in both groups.

## 4. Discussion

Based on the results of the phase III Checkmate 214 clinical trial, the combination of ipilimumab and nivolumab is a standard-of-care treatment for metastatic renal cell carcinoma. Elderly patients and those with poor performance status are generally excluded from clinical trials, despite them comprising a growing proportion of patients with metastatic RCC seen in clinical settings [19]. This lack of representation constitutes a challenge for physicians due to limited information regarding the efficacy and toxicity of ipilimumab and nivolumab in these patient populations. Retrospective studies have been conducted to try to answer these questions.

An analysis of the IMDC data found that patients over the age of 70 who received a programmed cell death PD(L)-1-based immune checkpoint inhibitor had no difference in OS or TTF but did have a lower overall response rate (ORR) when compared to younger adults [20]. A network meta-analysis of first-line and salvage treatments in metastatic RCC compared the efficacy of systemic therapies between older and younger patients [21]. It concluded that ipilimumab plus nivolumab was the most efficacious treatment for older patients, with improved PFS and OS; however, this efficacy was reduced compared to younger patients, and the specific toxicity profiles in the elderly population were not examined. In the Checkmate 214 study, 38% (323/847) of patients were 65 years old or greater, and less than 8% of patients were over the age of 75 (65/847). The hazard ratio (HR) for overall survival of the entire cohort, on the intention to treat analysis, was 0.66 (0.53–0.82). For patients < 65 years old, a hazard ratio of 0.53, with tight confidence intervals of 0.40–0.71, strongly favored ipilimumab and nivolumab over sunitinib. Subgroup analysis of overall survival for patients 65–75 years old and >75 years old favored ipilimumab and nivolumab over sunitinib (HR 0.86 and 0.97, respectively); however, the 95% confidence levels were wide (0.58–1.27 and 0.48–1.95, respectively), reflecting the small number of older patients participating in this study.

Our study analyzed real-world data from the CKCIS database of older patients with metastatic renal cell cancer who were >70 years of age and >75 years of age. We saw no impact of older age on survival outcomes or on toxicity in the multivariate analysis. We did see a difference in the median OS in the univariate analysis of patients age ≥ vs. <75 years old which was not statistically significant in the multivariate analysis, indicating that much of this prognostic difference is due to IMDC risk category distribution among these groups. Patients in our study who were older than 70 years and older than 75 years of age were significantly less likely to complete four cycles of ipilimumab and nivolumab, to have a nephrectomy, and to receive second-line treatment compared to younger counterparts. Our findings likely reflect differences between patients selected for clinical trials compared to a real-world population due to age, fitness, and comorbidities [7]. Our study indicates that in the real-world setting, older patients benefit from ipilimumab and nivolumab similarly to their younger counterparts.

In the comparison between patients age ≥70 vs. <70 years old, median PFS was worse in older adults (12.3 vs. 5.5 months, multivariate *p*-value 0.019), and median TTF tended to be worse in that population as well (15.2 vs. 11.7 months, multivariate *p*-value 0.077). Immunosenescence, defined as the deterioration of the immune system associated with age, might account for the reduced progression-free survival and time to treatment failure in our study. Due to the exclusion of elderly patients in clinical trials, the impact of immunosenescence on immunotherapy outcomes in the older population has not been fully explored [22,23].

There have been a few retrospective studies assessing the impact of performance status on outcomes in metastatic renal cell cancers. A retrospective analysis of a frontline immunotherapy-based combination treatment in patients with metastatic RCC and Eastern Cooperative Oncology Group (ECOG) performance status greater than or equal to 2 saw inferior survival outcomes compared to those described in the pivotal trials [24]. These data were echoed in a subgroup analysis of immunotherapy in patients with metastatic urothelial carcinoma [25]. We are already aware that KPS < 80 is a poor prognostic factor in the IMDC, and this has been validated prospectively [26]. In the Checkmate 214 study, the Karnofsky performance score had to be at least 70 to be eligible. In a subgroup analysis of the Checkmate 214 study, Karnofsky Performance Status (KPS) of less than or equal to 70 was associated with poorer outcomes, with a median OS of 16.8 months (N = 55) and 30 months OS of 32% compared to median OS not reached and 30 months OS of 68% (HR 2.8 CI 95% 1.93–4.06, *p* value < 0.0001) in those with a KPS greater than 70% [27].

Our real-world data from the national CKCis database supported prior data, showing that decreased performance status was significantly inferior with regard to median OS (multivariate *p*-value 0.0009), cause-specific survival (multivariate *p*-value 0.009), and median PFS (multivariate *p*-value 0.0007). Our data showed that patients with low KPS were significantly less likely to complete four cycles of ipilimumab and nivolumab, have a nephrectomy, and receive second-line treatment. The inability to complete four cycles of doublet immunotherapy could be a factor explaining the inferior outcomes in the patients with a poor performance status. This may also explain the decreased toxicity rates in patients with poor performance status because they received less treatment.

The patient demographic and clinical characteristics in our study show some differences compared to the pivotal Checkmate 214 study, which highlights the real-world nature of our study. As we have previously highlighted, the patients in our above cohorts had a nephrectomy (40–50%) compared to 80% in the Checkmate 214 trial [1]. Moreover, in our real-world data, only approximately half of the patient groups were able to complete four cycles of ipilimumab and nivolumab vs. 79% from the patients in the immunotherapy arm in Checkmate 214 [1]. The rate of treatment discontinuation in our cohort was also higher than the 22% treatment discontinuation rates reported in the Checkmate 214 trial. The rate of progressive disease in our trial was higher in the older and low-KPS population (at a range of 39–44%) compared to the Checkmate 214 trial (19%). These are important factors to take into consideration when deciding on first-line treatment. As clinicians, we need to consider the higher progressive disease rate in those patients when we decide on optimal first-line treatment.

Our study has some clear strengths. Our real-world data were obtained from a national database which includes patients from several provinces and varied healthcare centers and has been shown to be generalizable to the entire Canadian kidney cancer population [28]. Unfortunately, despite this, sample sizes for some groups were still quite small and groups were unbalanced, influencing the statistical power of this study. Another limitation of our study includes the fact that some data were missing and not accounted for in the national database. A future avenue for improvement in studies examining the effect of treatment on these populations is that they should include the use of patient-reported outcomes (PROs) [29]. In patients with metastatic disease, especially in older patients or those with poor performance status, the focus might be on health-related quality of life more so than survival benefits. The CKCis group is exploring the feasibility of collecting PROs within the current infrastructure.

Doublet immunotherapy is only one option for the first-line treatment of elderly and poor performance status patients with metastatic renal cell carcinoma. Our study results do not allow us to answer the question regarding optimal treatment choice between doublet immunotherapy or combination immunotherapy and vascular endothelial growth factor tyrosine kinase inhibitor for elderly or poor performance status patients. Studies are currently underway to attempt to answer this question in the elderly population.

Future prospective studies including older patients and those with poor performance status are required to appropriately answer these topical questions. The RAMONA study, a one-of-its-kind prospective phase II study evaluating dual immunotherapy in an elderly population with advanced esophageal squamous cell cancer, is leading the way in the use of geriatric screening tools to assess for trial eligibility in this population [30].

## 5. Conclusions

Our results show that older age is not associated with inferior OS or increased toxicity. In contrast, patients with poor performance status have inferior outcomes when treated with a combination of ipilimumab and nivolumab. Our study revealed higher rates of primary progression in patients with lower performance status and older age; it is unclear if this is simply prognostic or predictive of the benefit of dual immunotherapy. In our daily practice, we need to consider these outcomes when planning and discussing our treatment options with patients.

## Figures and Tables

**Table 1:** Baseline characteristics and outcomes age <70 with age ≥70.

	Age < 70 N = 415	Age ≥ 70 N = 136	*p*-Value
Median Age (years)	60.6	74.4	
Sex	N = 415	N = 136	
Male N (%)	307 (74.0)	100 (73.5)	0.918
Female N (%)	108 (26.0)	36 (26.5)	
IMDC Risk	N = 342	N = 112	
IMDC favorable N (%)	29 (8.5)	6 (5.4)	0.544
IMDC intermediate N (%)	202 (59.1)	67 (59.8)	
IMDC poor N (%)	111 (32.5)	39 (34.8)	
Cycles of Ipi/Nivo received	N = 415	N = 136	
Completed 4 cycles of Ipi/Nivo N (%)	250 (60.2)	60 (44.1)	0.007
Surgical Resection	N = 415	N = 136	
Nephrectomy N (%)	283 (68.2)	81 (59.6)	0.065
Metastasectomy N (%)	91 (21.9)	20 (14.7)	0.068
Outcomes	N = 414	N = 136	
Median OS (mo)	52.1	45.1	0.343 (0.352 *)
Median CSS (mo)	93.3	NR	0.830 (0.897 *)
Median PFS (mo)	12.3	5.5	0.013 (0.019 *)
Median TTF (mo)	15.2	11.7	0.048 (0.077 *)
Toxicity	N = 415	N = 136	
Toxicity (%)	163 (39.3)	54 (39.7)	0.929
Response rates	N = 368	N = 112	
CR N (%)	27 (7.3)	7 (6.3)	0.972
PR N (%)	122 (33.2)	36 (32.1)	
SD N (%)	80 (21.7)	25 (22.3)	
PD N (%)	139 (37.8)	44 (39.3)	
	N = 415	N = 136	
Received second-line treatment N (%)	201 (48.4)	51 (37.5)	0.026

mo = months, OS = overall survival, CSS = cause-specific survival, PFS = progression-free survival, TTF = time to treatment failure, NR = not reached, CR = complete response, PR = partial response, SD = stable disease, PD = progressive disease, * multivariate analysis.

**Table 2:** Baseline characteristics and outcomes age <75 with age ≥75.

	Age < 75 N = 494	Age ≥ 75 N = 57	*p*-Value
Median age (years)	61.9	77.7	
Sex	N = 494	N = 57	
Male N (%)	365 (73.9)	42 (73.7)	0.974
Female N (%)	129 (26.1)	15 (26.3)	
IMDC Risk	N = 406	N = 48	
IMDC favorable N (%)	32 (7.9)	3 (6.3)	0.758
IMDC intermediate N (%)	242 (59.6)	27 (56.3)	
IMDC poor N (%)	132 (32.5)	18 (37.5)	
Cycles of Ipi/Nivo received	N = 494	N = 57	
Completed 4 cycles of IPI NIVO	284 (57.5)	26 (45.6)	0.086
Surgical Resection	N = 494	N = 57	
Nephrectomy N (%)	335 (67.8)	29 (50.9)	0.011
Metastasectomy N (%)	103 (20.9)	8 (14.0)	0.225
Outcomes	N = 493	N = 57	
Median OS (mo)	53.7	30.0	0.032 (0.157 *)
Median CSS (mo)	93.3	45.3	0.127 (0.320 *)
Median PFS (mo)	10.2	6.6	0.287 (0.386 *)
Median TTF (mo)	13.9	14.6	0.444 (0.469 *)
Toxicity	N = 494	N = 57	
Toxicity (%)	195 (39.5)	22 (38.6)	0.898
Response Rates	N = 431	N = 49	
CR N (%)	31 (7.2)	3 (6.1)	0.778
PR N (%)	141 (32.7)	17 (34.7)	
SD N (%)	92 (21.4)	13 (26.5)	
PD N (%)	167 (38.8)	16 (32.7)	
	N = 494	N = 57	
Received second-line treatment N (%)	234 (47.4)	18 (31.6)	0.024

mo = months, OS = overall survival, CSS = cause-specific survival, PFS = progression-free survival, TTF = time to treatment failure, CR = complete response, PR = partial response, SD = stable disease, PD = progressive disease, * multivariate analysis.

**Table 3:** Baseline characteristics and outcomes for KPS ≥ 70 with KPS < 70.

	KPS ≥ 70 N = 448	KPS < 70 N = 63	*p*-Value
Median age (years)	63.4	64.7	
Sex	N = 448	N = 63	
Male N (%)	338 (75.5)	38 (60.3)	0.011
Female N (%)	110 (24.6)	25 (39.7)	
IMDC risk	N = 395	N = 52	
IMDC favorable N (%)	35 (8.9)	0	<0.0001
IMDC intermediate N (%)	253 (64.1)	10 (19.2)	
IMDC poor N (%)	107 (27.1)	42 (80.8)	
Number of cycles of ipi/nivo completed	N = 448	N = 63	
Completed 4 cycles of Ipi/Nivo	266 (59.4)	23 (36.5)	<0.0004
Surgical Resection	N = 448	N = 63	
Nephrectomy N (%)	303 (67.6)	27 (42.9)	<0.0001
Metastasectomy N (%)	89 (19.9)	12 (19.1)	0.879
Outcomes	N = 447	N = 63	
Median OS (mo)	54.5	10.8	<0.0001 (0.0009 *)
Median CSS (mo)	NR	14.3	<0.0001 (0.009 *)
Median PFS (mo)	11.6	3.1	<0.0001 (0.0007 *)
Median TTF (mo)	15.2	7.2	0.001 (0.123 *)
Toxicity	N = 448	N = 63	
Toxicity (%)	184 (41.1)	16 (25.4)	0.017
Response rates	N = 394	N = 51	
CR N (%)	27 (6.9)	5 (9.8)	0.508
PR N (%)	135 (34.3)	13 (25.5)	
SD N (%)	84 (21.3)	10 (19.6)	
PD N (%)	148 (37.6)	23 (45.1)	
	N = 448	N = 63	
Received second-line treatment N (%)	218 (48.7)	18 (28.6)	0.003

mo = months, OS = overall survival, CSS = cause-specific survival, PFS = progression-free survival, TTF = time to treatment failure, NR = not reached, CR = complete response, PR = partial response, SD = stable disease, PD = progressive disease, * multivariate analysis.

## Data Availability

The datasets presented in this article are not readily available as this is a registry that includes real-world data that are not available to the public.
